# The Incidence of Osteoporosis in Hepatocellular Carcinoma Patients Under 65: A Retrospective Cohort Study

**DOI:** 10.7759/cureus.80490

**Published:** 2025-03-12

**Authors:** Katherine Quesada Tibbetts, Rahul Mhaskar, Neelesh Prakash

**Affiliations:** 1 Radiology, University of South Florida Health Morsani College of Medicine, Tampa, USA; 2 Internal Medicine, University of South Florida Health Morsani College of Medicine, Tampa, USA

**Keywords:** bone demineralization, cancer, ct, dexa, endocrinology, hepatocellular carcinoma, imaging guidelines, liver disease, osteoporosis, preventative medicine

## Abstract

Introduction: Hepatocellular carcinoma (HCC) patients have a heightened prevalence of low bone mineral density (BMD) and the development of osteoporosis. Osteoporosis screening guidelines only recommend dual-energy X-ray absorptiometry (DEXA) scans for females 65 and older and males 70 and older. We set out to analyze the incidence of low BMD in HCC patients under 65 years old and encourage implementation of DEXA screenings for this patient population.

Method: In this retrospective cohort study, 170 patients under 65 with an HCC diagnosis were analyzed. Hounsfield units (HU) from L1 non-contrast CT scans are a reliable predictor of T-scores from DEXA scans and were used to predict BMD in patients, with scores of less than 165 HU indicative of osteopenia and less than 98 indicative of osteoporosis.

Results: The median HU score of patients was 137.2, and the mean score was 142.6 (minimum: 55.4; maximum: 303.1). Of the total 170 patients, 128 (75%) had an HU score of less than 165, indicating a high likelihood of suffering from low BMD. Among low BMD patients, 25 (20%) were identified as within a range of osteoporosis.

Conclusions: HCC patients under 65 have an increased incidence of bone demineralization. We suggest that BMD in HCC patients is an important prognostic tool and parameter to document, as studies have shown that HCC patients with high BMD have longer overall survival than patients with low BMD. Future prospective studies using DEXA scans to assess BMD should be completed to verify the risk of osteoporosis.

## Introduction

Hepatocellular carcinoma (HCC) is one of the leading causes of cancer-related deaths in the United States. Often a result of chronic liver injury, HCC in patients has been linked to several nutritional deficiencies which have adverse health effects on patients and prognosis [1-3]. Among these risks in HCC patients is a heightened risk for low bone mineral density (BMD) and the development of osteoporosis, which can lead to increased fracture risk and joint inflammation [2,3]. The risk of osteoporosis or fracture is higher in patients with liver disease than in the general population [4]. It has been shown that patients with chronic liver disease suffer from vitamin deficiencies, which lead to decreased bone synthesis and a higher risk of low BMD. Additionally, studies have shown that chronic liver disease patients with low BMD have an increased risk of developing HCC [1,5]. For example, patients with liver cirrhosis often develop vitamin D deficiency, which further predisposes them to the development of HCC [1]. This data suggests that many HCC patients are at an increased risk for these same nutritional deficiencies and low BMD [5].

We suggest that BMD is an important prognostic tool and parameter to document in patients who have been diagnosed and are undergoing treatment for HCC. Studies have shown that HCC patients with high BMD have more prolonged overall survival than patients with low BMD [6]. Additionally, HCC patients with low BMD had worse survival rates post liver transplant than patients with normal BMD [1,6,7]. Furthermore, osteoporosis is already a known complication of liver transplantation, and the younger patient population of HCC patients may be at increased risk of premature bone demineralization [8]. Thus, these measurements are important for HCC treatment plans.

Dual-energy x-ray absorptiometry (DEXA) scans are the most reliable method of assessing BMD in patients and are regarded as the gold standard [9-13]. However, current screening guidelines recommend DEXA scans for osteoporosis in females 65 and older and males 70 and older; earlier screening is recommended only in patients under 65 with one or more predefined risk factors (history of fractures, low body weight, smoking, or glucocorticoid therapy), which currently do not include cancer history [14,15]. Given the adverse effects and implications of low BMD in HCC patients, we believe young HCC patients under 65 must receive DEXA osteoporosis screenings if this population shows low BMD.

In this study, we used existing L1 CT scans as an alternative indicator of osteopenia or osteoporosis in HCC patients under 65 to analyze the prevalence of low BMD in this population. The use of Hounsfield units (HU) from CT scans is a reliable predictor of T-scores from DEXA scans and can be used to predict osteoporosis risk in patients [16-20]. Researchers have shown that the CT attenuation threshold of an L1 vertebra of less than 160 HU has a 90% sensitivity to osteoporosis as evaluated with DEXA [9]. Yang et al. have described research values for bone demineralization using HU at the L1 vertebra. In their study, researchers found a level of 165 HU to be the threshold of normal BMD, with less than indicative of osteopenia, and less than 98 indicative of osteoporosis [18].

Given the prevalence of nutritional deficiencies and the probability of osteoporosis in HCC patients under 65, this population may benefit from regular DEXA screenings to detect low BMD early and provide effective treatment before further damage ensues. In this study, we analyzed the prevalence of low BMD in HCC patients under 65 to assess the incidence of low BMD by analyzing CT scans at the L1 vertebra on the HU scale. Considering the bone loss, high tumor burden, and prevalence of liver transplantation in HCC patients, we believe the results of this study can further improve prognostic knowledge, imaging guidelines, and treatment plans for HCC patients under 65.

This article was previously presented as a meeting abstract at the 2024 Canadian Association of Radiologists Annual Scientific Meeting between April 11 and 14, 2024. This article was previously posted to the Research Square preprint server on September 2, 2024.

## Materials and methods

Study design

Patients

This retrospective cohort study qualified for a waiver of the requirement for signed authorization from the Institutional Review Board (IRB) as outlined in the Health Insurance Portability and Accountability Act (HIPAA) Privacy Rule regulations at 45 Code of Regulations (CFR) 164.512(i). We conducted a retrospective chart and radiographic review that evaluated abdominal CT scans without contrast for all patients under 65 years of age diagnosed with HCC at the Tampa General Hospital (FL, USA) between January 1, 2019, and January 1, 2021. The inclusion criteria were any patient under 65 years of age who was diagnosed in our study time frame and had an abdominal non-contrast CT scan on file. There were no exclusion criteria. Demographic information and medical history were collected.

Image Acquisition and Interpretation

The non-contrast abdominal CT scans were acquired from patient charts during our study time frame. To minimize bias, the first non-contrast CT scan from diagnosis of HCC was utilized for measurements. The CT scans were analyzed at the L1 vertebra with HU to predict BMD. The HU measurement tool on the Picture Archiving and Communication System was utilized to take the measurements at the level of the L1 vertebra.

Reference Standard

Given the study design as a retrospective cohort study, there was no control group. The HU results were analyzed against criteria outlined by Yang et al. According to their research, HU analysis at the L1 vertebra of less than 165 is indicative of osteopenia and less than 98 is indicative of osteoporosis [18].

Statistical analyses

We conducted the descriptive analysis to report frequencies for categorical data and mean and standard deviation (SD) or median, minimum, and maximum for continuous data. We reviewed the difference in the distribution of continuous variables across categorical variables using the Mann-Whitney U and Kruskal-Wallis test. We investigated the correlation by Spearman's rho (r) and reported the correlation coefficient (r) and the two-tailed 95% confidence intervals (95% CIs). For additional analyses, we created two groups of patients based on the 165 cut off for HU data. All analyses were conducted by SPSS Statistics version 26 (IBM Corp., Armonk, NY, USA). A p-value less than 0.05 denotes statistical significance.

## Results

**Figure 1:**
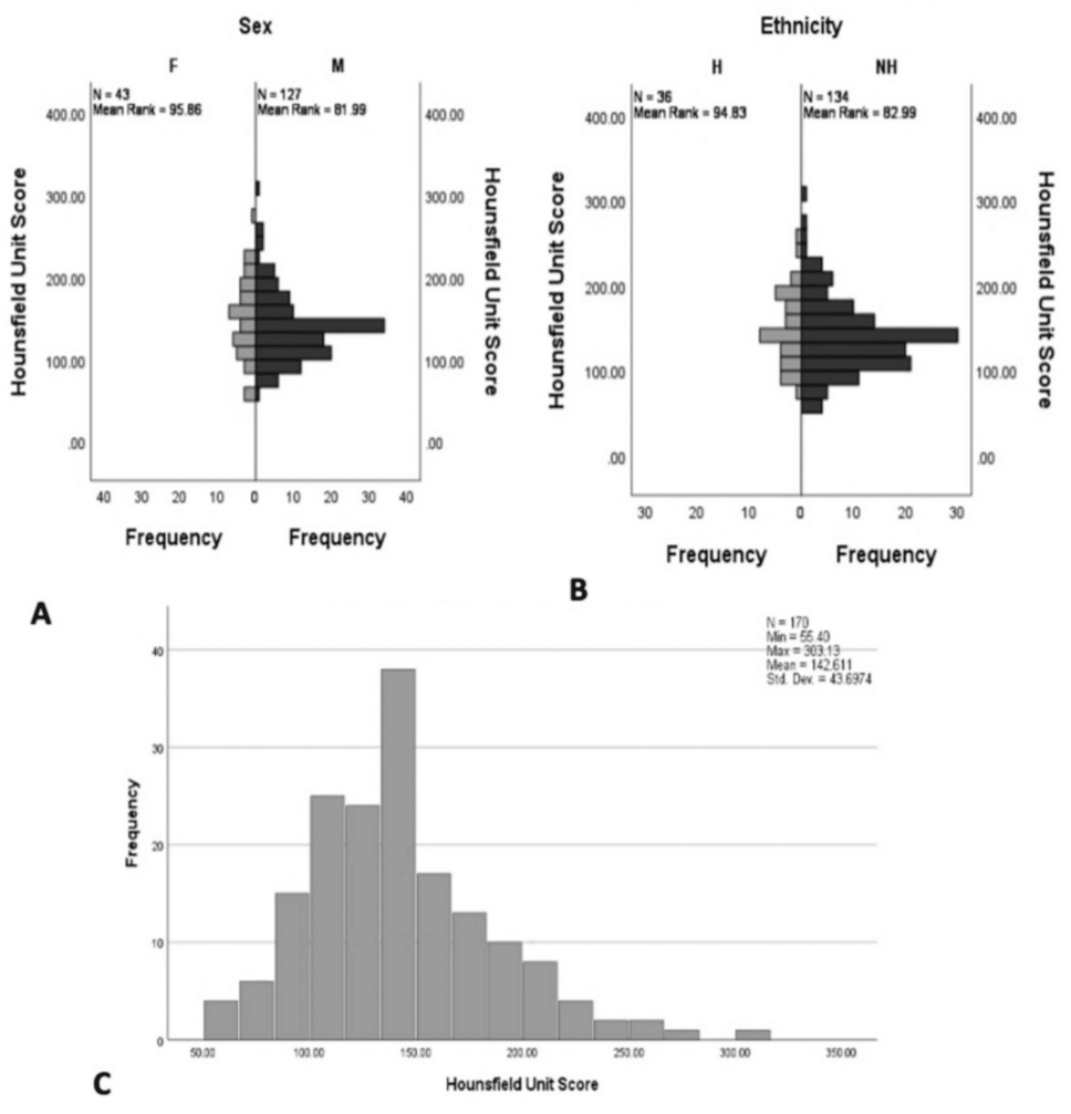
Demographic analysis of HU A: Distribution HU Scores based on sex; B: HU Scores based on ethnicity; C: Frequency bar graph for all HU data combined HU: Hounsfield units, H: Hispanic, NH: Non-hispanic

A total of 170 patients met the inclusion criteria of being under 65 years of age with a diagnosis of HCC and having a non-contrast abdominal CT scan over a two-year period. The average patient in the cohort was Caucasian, non-Hispanic, and male, with an average age of 58.3 years (Figure 1A and 1B). The BMI was recorded for the patient cohort, with a median of 27.0 kg/m2 (min: 10.1, max: 72.45) and a mean of 28.4 kg/m2 (SD: 7.41). The median age was 60 years (min: 26.0, max: 64.0), with a mean of 58.4. The median HU score for the patient cohort was 137.2 HU, with a minimum of 55.3 HU and a maximum of 303.1 HU (Figure 1C). There were 43 (25%) females and 127 (75%) males. For females, the median HU was 152.0 (min: 55.4, max: 274.9). For males, the median HU was 136.5 (min: 65.9, max: 303.1). The patient cohort consisted of 142 (83.5%) Caucasian, 17 (10%) Black, three (2%) Asian, one (0.5%) Arab, and six (3.5%) Native American patients (Table 1). The cohort included 36 (21.2%) Hispanic patients with median HU of 143.4 (min: 81.0, max: 258.1). Of the patients, 128 (75%) were below the threshold of 165 HU and, therefore, had a potential for an increased probability of low BMD. Of these low BMD patients, approximately 25 (20%) were identified within the range of osteoporosis. In the full cohort, these 25 (15%) patients were identified as potentially having osteoporosis if evaluated by DEXA.

**Table 1:** Comparison of HU scores across race HU: Hounsfield units

Race	N	Median	Minimum	Maximum
Arab	1	124.7	124.7	124.7
Asian	3	146.6	145.1	151.7
Black	17	188.9	128.5	303.1
Caucasian	142	134.4	55.4	258.1
Native American	6	149.6	90.7	191.4
Total	169	137.2	55.4	303.1

**Figure 2:**
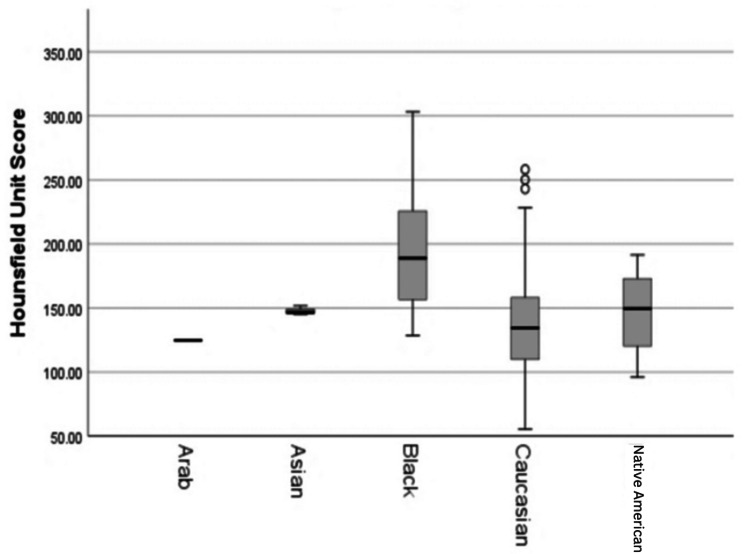
Relationship between race and HU score This graph illustrates that Caucasians had lower HU scores on average. HU: Hounsfield units

The Charlson co-morbidity index was calculated for each patient and was distributed evenly across patients based on their HU score grouping based on the 165 cut off (p = 0.192). In our cohort, Caucasians were most at risk for low BMD (median HU score: 134.4, min: 55.4, max: 258.1) (Figure 2). The HU score had a significant negative correlation to glucose (r = -0.159, 95% CI (-0.306, -0.004), p = 0.038), age (r = -2.00, 95% CI (-0.344, -0.046), p = 0.009), and potassium (r = -0.275, 95% CI (-0.413, -0.126), p < 0.001). The median albumin score for the patient cohort was 3.3 g/dL (min: 1.1, max: 4.9). The HU did not have a significant relationship to albumin (r = -0.128, 95% CI [-0.278, 0.027], p = 0.095) or BMI (r = -0.030, 95% CI (-0.184, 0.125), p = 0.695). Only 33 patients had a vitamin D level measurement in their medical chart, and the median vitamin D level was 16.0 ng/mL (min: 3.5, max: 69.0).

Analysis of the patients under vs. over 165 HU was also completed (Table 2). Overall, the data for those at risk of low BMD (<165 HU), has statistically significant results for potassium (p = 0.004) and age (p = 0.022). Non-statistically significant relationships included BMI (p = 0.233), Charlson co-morbidity index (p = 0.192), glucose (p = 0.446), vitamin D level (p = 0.505), and albumin (p = 0.156).

**Table 2:** Comparison of variables between less than 165 HU and greater than 165 HU score *For vitamin D analysis: <165, n = 23; >165, n = 10; total, n = 33 **Significant value HU: Hounsfield units

Variables	Less than 165 (n=128)			Greater than 165 (n=42)			Total (n=140)			p-value
	Median	Minimum	Maximum	Median	Minimum	Maximum	Median	Minimum	Maximum	
Age (years)	60.00	29.00	64.00	58.00	26.00	64.00	60.00	26.00	64.00	0.022**
BMI (kg/m^2^)	27.22	10.07	72.45	26.24	17.90	49.25	26.99	10.07	72.45	0.23
Vit D (ng/mL)*	16.20	10.07	29.11	13.90	3.40	69.00	16.00	3.40	69.00	0.51
Glucose (mg/dL)	106.50	72.00	320.00	103.50	70.00	399.00	105.50	70.00	399.00	0.45
Potassium (mmol/L)	4.30	3.10	10.60	4.00	2.90	4.90	4.20	2.90	10.60	0.004**
Albumin (g/dL)	3.30	1.10	4.90	3.20	1.20	4.60	3.30	1.10	4.90	0.16

## Discussion

**Figure 3:**
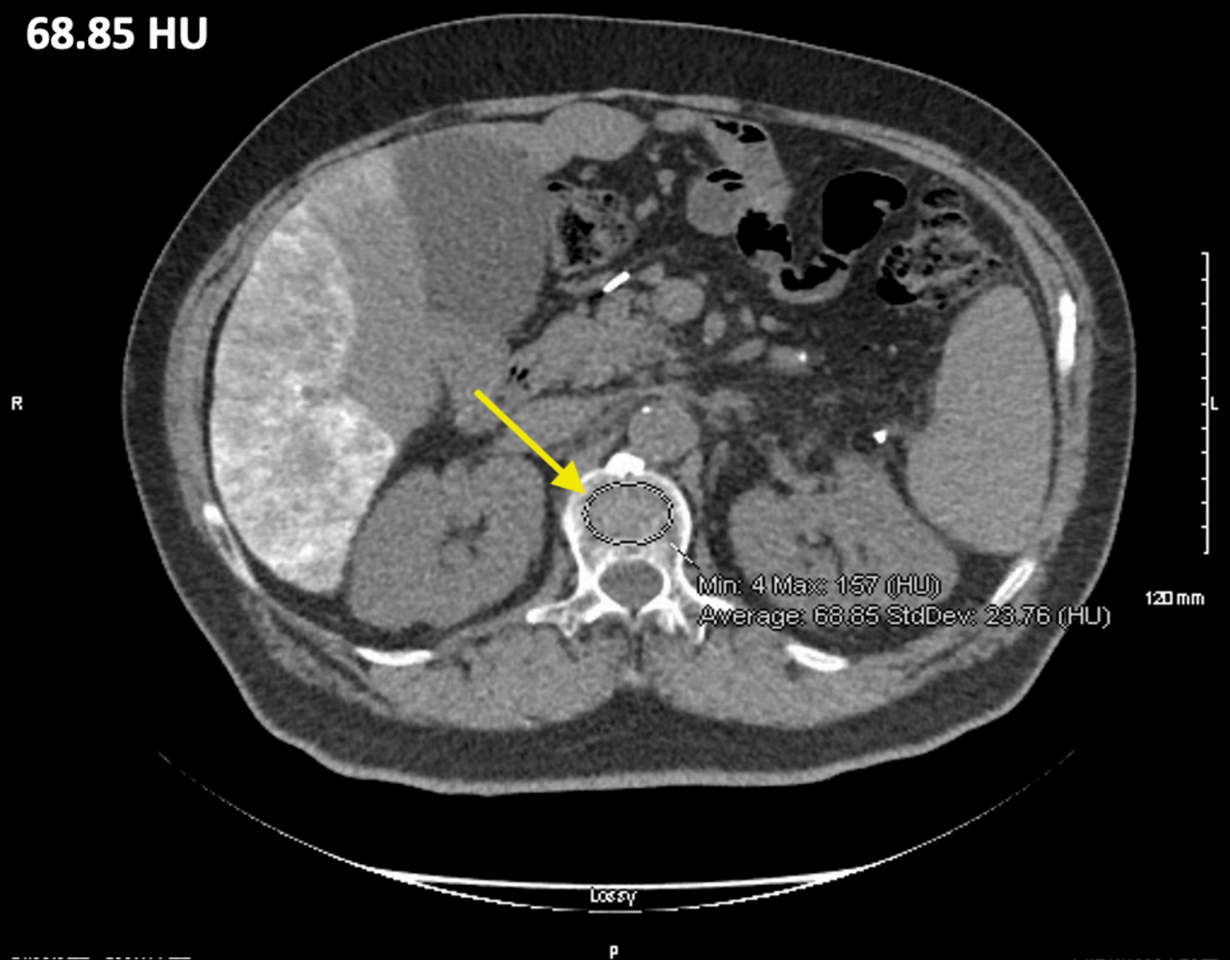
L1 Vertebra of an HCC patient The unenhanced axial abdominal CT scan of the L1 vertebra (arrow) in a 58-year-old male patient with HCC shows a measurement of 68.85 HU. HCC: Hepatocellular carcinoma, HU: Hounsfield units

As shown by the results of this study, HCC patients are at risk of low BMD due to their liver disease and nutritional deficiencies. The technique of using HU on non-contrast CT scans to identify high risk HCC patients for prematurely developing osteopenia or osteoporosis is a useful application of this modality (Figure 3) [21-24]. With 128 (75%) patients from the study below the 165 HU threshold for normal BMD, we recommend that HCC patients undergo regular DEXA screening to assess individual risks for osteopenia or osteoporosis and the associated complications. Measurements for the nutritional deficiencies of the patient cohort were evaluated by the measurement of albumin, vitamin D, glucose, and BMI.

Most of the cohort had an overweight BMI, indicating a serious co-morbidity. Although albumin did not have a statistically significant relationship with HU score, the median and mean albumin levels were below the normal range, indicating that the patient cohort experienced nutritional deficiencies, as expected, given the liver disease. Vitamin D levels were also consistently below normal values, strengthening the argument for better nutritional management of HCC patients [3]. Surprisingly, there was a significant inverse relationship between HU score and glucose level, indicating that hyperglycemia is associated with lower BMD and, thus, worse prognostic factors.

The under 165 HU subgroup analysis was not significant for a relationship between HU and glucose, indicating once low BMD is acquired, glucose levels do not indicate severity of osteopenia. However, among whole data from the HCC cohort patients, hyperglycemia was a significant predictor of having an increased risk of low BMD. This analysis shows that hyperglycemia may be a predictor of the heightened risk of developing low BMD in HCC patients. However, further investigation into the relationship between glucose and BMD is needed to fully understand the implications of hyperglycemia in this patient population.

Inherent limitations are present given the study design as a retrospective chart review. Limitations of this study include the varied time between cohort members from point of diagnosis to point of first non-contrast CT scan and variability in the CT machine used. These were uncontrollable variables given the nature of the retrospective study. Researchers attempted to limit time variability between individual patient CT scans and disease progression by recording HU scores from the first CT scan without contrast on the patient record after diagnosis. 

With the nutritional markers and imaging results considered, it is evident that HCC patients suffer from declining nutritional values and increasing co-morbidities, which may have negative implications on their disease prognosis and risk of developing low BMD and the associated complications [3]. Future research into cirrhotic patients is also recommended considering the clinical importance of BMD in liver disease patients as a predictor of HCC [1,25,26]. As described by Liang et al., BMD is an effective predictor of the development of HCC in cirrhotic patients [1].

With current DEXA screening guidelines recommending imaging for women 65 and older and men 70 and older, a sizable subset of cancer patients are left with increased risk of unmonitored early-onset bone demineralization and are left to its consequences [14]. Through this study, it is evident HCC patients under 65 years old have a high incidence of low BMD and an increased fracture risk, and physicians should consider regular BMD screenings in this patient cohort. The Endocrine Society clinical guidelines recommend DEXA screenings of younger patients if they are shown to be at heightened risk of bone demineralization [15]. The results of this study identify HCC patients under 65 as high-risk patients, and future studies utilizing DEXA scans should be completed to determine appropriate screening guidelines for this patient population.

## Conclusions

In conclusion, HCC patients under 65 were found to have low BMD, as assessed by HU scores at the L1 level in non-contrast CTs. Of the 170 HCC patients assessed, 128 (75%) were recorded to have low BMD. Our data supports the potential benefit of regular DEXA scans for this patient population in order to carefully screen for the development of osteopenia and osteoporosis and to avoid further complications in their care and management. Given this patient cohort was found to have poor nutritional markers, nutritional supplementation and aid must be provided to improve prognosis and mitigate unnecessary risk factors. Hyperglycemia and low BMD share a statistically significant inverse relationship, and future research is encouraged to investigate further the influence hyperglycemia can have on BMD. Given the findings of our study, we recommend that future research, such as a prospective DEXA scan study, be performed to evaluate the clinical effectiveness of BMD screening in HCC patients and determine appropriate screening recommendations.
